# Integrative machine learning and Mendelian randomization identify causal laboratory biomarkers for coronary artery lesions in Kawasaki disease: a prospective study

**DOI:** 10.3389/fgene.2025.1646032

**Published:** 2025-08-15

**Authors:** Hancao Yang, Meng Wu, Keqing Liang, Yi Li, Ran Yang, Beibei Yuan, Ming Wu, Jin Xu

**Affiliations:** 1 Department of Clinical Laboratory, Children’s Hospital of Fudan University & National Children Medical Center, Shanghai, China; 2 Department of Clinical Laboratory, Children’s Hospital of Nanjing Medical University, Nanjing, China; 3 Department of Pediatric Surgery, Children’s Hospital of Fudan University & National Children Medical Center and Shanghai Key Laboratory of Birth Defect, Shanghai, China

**Keywords:** Kawasaki disease, coronary artery lesions, machine learning, Mendelian randomization, laboratory biomarkers

## Abstract

Kawasaki disease (KD) patients could develop coronary artery lesions (CALs) which threatens children’s life. We aimed to develop and validate an artificial intelligence model that can predict CALs risk in KD patients. A total of 506 KD patients were included at Children’s Hospital of Fudan University. Seven predictive features were identified for model building. Among different machine learning (ML) models tested, Multi-Layer Perceptron Classifier (MLPC), Random Forest (RF) and Extra Tree (ET) demonstrated optimal performance. These were finally chosen for time-across validation. Among three of them, MLPC stands out with its highest accuracy. Besides, Mendelian randomization (MR) analysis also provided genetic evidence. Among seven predictive features, two of them were identified as causal associations with CALs. They are activated partial thromboplastin time (APTT) and red cell distribution width (RDW). The causal mechanism reinforced the biological plausibility of the model. ML-based prediction models, combined with genetic validation through MR, offer a reliable approach for early CALs risk stratification in KD patients. This strategy may facilitate timely clinical interventions.

## 1 Introduction

Kawasaki Disease (KD), first described by Tomasaku Kawasaki in 1967 (Noval Rivas and Arditi, 2020), is one of the most common forms of vasculitis in childhood. It is usually a self-limited disorder and, if left untreated, fever and other manifestations of acute inflammation last an average of 12 days. KD mostly affecting medium and large-sized vessels particularly coronary arteries, and finally leading to coronary artery lesions (CALs) (Noval Rivas and Arditi, 2020; Saadoun et al., 2021). KD can cause a variety of cardiovascular complications, including coronary artery aneurysms, cardiomyopathy with decreased myocardial contractility and heart failure, myocardial infarction, arrhythmias, and peripheral artery occlusion. 25% of patients with KD have developed CALs, which is the leading cause of acquired cardiac disease in children (Platt et al., 2020). As the major complication of KD, CALs include several syndromes, such as arrhythmias, acute coronary syndrome, and pericarditis and/or myocarditis-like syndromes. These complications can lead to serious morbidity and even death. Therefore, the most important aspect of KD is the prevention of CALs.

With the widespread of intravenous immunoglobulin (IVIG) therapy around the world, the prevalence of CALs in KD patients has been significantly reduced, but CALs still occur in 5%–20% patients with KD in the acute phase (Makino et al., 2019; Skochko et al., 2018). Early diagnosis of CALs is very important as it allows performing appropriate disease management and treatment. So far, imaging methods that are invasive (coronary angiography, intracoronary ultrasound) are accurate to assess coronary disease. However, its invasiveness, radiation exposure and high technical requirements limit its application. More practical and convenient options for patients are needed. In recent years, more and more research has been carried out on other influencing factors of CALs complicated by KD in the world (Noval Rivas and Arditi, 2020). Some parameters such as D-dimer, C-reactive protein (CRP), platelets, neutrophil aggregates and inflammatory cytokine levels have been reported as biomarkers for predicting CALs (Lam et al., 2022; Kostik et al., 2021). But the underlying pathogenesis of CALs with KD is largely unknown. Therefore, further investigations of the risk factors of CALs are highly warranted in KD patients.

Machine learning (ML), one of the major building blocks of artificial intelligence (AI), has been applied in many different fields and has shown great potential in assisting clinical diagnosis (Greener et al., 2022; Handelman et al., 2018). Scholars from various countries have used different algorithms to predict the risk of different diseases. With the development of the research, the definition and standard of CAL is becoming more and more refined. Therefore, previously established risk scoring systems (e.g., the Formosa scoring system, the Egami scoring system, and the statistical model advanced by the Kobayashi scoring system) are not particularly ideal in China (Kobayashi et al., 2006; Egami et al., 2006). There is an urgent need for a method to help predict that those high-risk children are prone to CALs. In 2016, through a study of large cohort data from the latest follow-up, Professor Gu Dongfeng’s team created the China-PAR model to assess the 10-year risk and lifetime risk of cardiovascular disease (Jiang et al., 2023). This model can predict the risk of different genders, and provides an effective tool for improving the level of primary protection and management of cardiovascular diseases. Similarly, ML has the potential to aid in early detection of CALs by modelling the complex relationships between clinical variables, but, to the best of our knowledge, there is currently no machine-learning algorithm that differentiates CALs from Kawasaki disease.

The role of laboratory parameters in KD remains unclear and evidence from observational studies may be subject to confounding and selection bias (Kelly et al., 2017). Mendelian randomization (MR) may provide unconfounded estimates. To clarify the role of influencing factors in CALs, we conducted a two-sample univariable MR study to assess the associations of possible indicators with KD using the largest and most recent genome wide associations studies (GWAS) (Burgner et al., 2009; Kim et al., 2011; Tsai et al., 2011; Khor et al., 2011; Onouchi et al., 2012; Lee et al., 2012; Kim et al., 2017). In response to the difficulty clinicians have in diagnosis of and differentiation between CALs and Kawasaki disease, we aimed to develop and validate a clinical decision support system to distinguish among children with or without CALs from Kawasaki disease, characterized by similar clinical and laboratory features in the early time.

## 2 Materials and methods

### 2.1 Participants

In this study, a total of 506 pediatric patients diagnosed with KD between February 2013 and November 2023 were enrolled at the Children’s Hospital of Fudan University. Venous blood samples were collected from KD patients at the time of initial evaluation in hospital. The blood analysis of the samples was conducted in the laboratory department of our hospital. The demographic and laboratory data were extracted from the medical record. All patients met the diagnostic criteria outlined in the Expert Consensus on the Diagnosis and Acute-Phase Treatment of Kawasaki Disease (Subspecialty Group of Rheumatology, 2022), which include persistent fever for ≥5 days and at least four of the five principal clinical features: polymorphous rash, bilateral nonexudative conjunctival injection, changes in lips and oral cavity, changes in the extremities, and cervical lymphadenopathy. The diagnosis of CALs was established based on echocardiographic findings, defined as a Z score ≥2 mm (Kuo, 2023).

Patients were excluded if they had received immunosuppressive therapy within the previous 3 months or had evidence of cardiac, hepatic, or renal insufficiency; active infections; or immunodeficiency disorders. All diagnoses and treatment decisions were made by one of two experienced pediatric clinicians specializing in KD. Detailed demographic and clinical characteristics of the enrolled patients are presented in Table 1.

**TABLE 1:** Dataset review.

Personal information	Data for ML model (n = 432)		Data for time validation (n = 74)	
	KD without CALs (n = 331)	KD with CALs (n = 101)	KD without CALs (n = 38)	KD with CALs (n = 36)
Gender (n)				
Male	199	80	28	28
Female	132	21	10	8
Age (n, year)				
0∼1	62	19	6	2
1∼3	133	24	13	7
3∼5	75	21	8	4
5∼7	33	15	4	2
7∼10	24	10	6	10
10∼18	4	12	1	11

### 2.2 Data preprocessing

For a fair comparison of performance across different input feature sets, rigorous data preprocessing procedures were implemented using the scikit-learn library in Python (version 3.9.13). Missing values in continuous variables were imputed using median substitution, a robust univariate method that reduces sensitivity to outliers while preserving the central tendency of the data. Continuous features were standardized using z-score normalization (mean = 0, standard deviation = 1) to ensure comparability across variables and to enhance algorithmic convergence. Where applicable, categorical variables were transformed using one-hot encoding to enable compatibility with machine learning models.

Following preprocessing, the dataset was randomly partitioned into training and testing subsets in an 80:20 ratio. Stratified sampling was employed to maintain consistent class distributions across subsets, thereby minimizing potential sampling bias due to class imbalance. All preprocessing steps were conducted prior to model training and cross-validation to prevent data leakage and ensure methodological rigor.

### 2.3 Feature selection

We employed five distinct algorithms to identify the most informative predictors from a large pool of candidate variables: Corrected as Gradient Boosting Decision Tree (GBDT), Extra Tree (ET), Random Forest (RF), Logistic Regression (LR), and Least Absolute Shrinkage and Selection Operator (LASSO) regression. These algorithms were selected for their capacity to rank feature importance based on different theoretical foundations—tree-based ensemble methods, linear coefficients, and regularization penalties. The aim was to adopt a data-driven approach that retains features with high predictive value while eliminating redundant or irrelevant variables, thereby enhancing model stability and performance on unseen data.

### 2.4 Model development

To develop predictive models for the diagnosis of CALs in KD patients, we implemented eight distinct ML algorithms: Support Vector Classifier (SVC), LR, RF, ET, Decision Tree Classifier (DT), Gaussian Naïve Bayes (Gaussian NB), K-Nearest Neighbors (KNN), and Multi-Layer Perceptron Classifier (MLPC). These algorithms were selected to represent a diverse range of classification paradigms, encompassing linear models, ensemble methods, probabilistic models, distance-based learning, and neural networks. All selected models are widely used in biomedical research and offer complementary strengths. Each algorithm was chosen for its balance between interpretability and capacity to capture complex linear or nonlinear relationships among input features.

### 2.5 Model evaluation

To assess the models’ discriminative power, receiver operating characteristic (ROC) curves were plotted, and the area under the curve (AUC) was calculated. Mul-tiple evaluation metrics were computed to comprehensively assess classification performance, including precision, recall, accuracy, and F1-score, all derived from the confusion matrix.

To rigorously evaluate model performance and minimize the risk of overfitting, stratified 10-fold cross-validation was conducted on the training dataset ensuring class balance across folds. Average performance across all folds was reported to ensure robustness and generalizability of the models. Model calibration was assessed by generating calibration (reliability) curves, and the Brier score (BS) was computed as a quantitative measure of the accuracy of probabilistic predictions. Lower BS indicate better calibrated models. In addition, the Kolmogorov–Smirnov (KS) test was applied to evaluate the separation between predicted probability distributions of the positive and negative classes.

Finally, to evaluate the temporal robustness and real-world applicability of the developed models, external validation was performed using a temporally independent test cohort collected after the model development period. This prospective validation strategy provided further evidence of the model’s generalizability to future clinical data.

### 2.6 Mendelian randomization analyses

Summary data on outcomes were collected from published GWAS meta-analyses and publicly available data. These summary data were analyzed by MR to determine if there was a causal association between selected features and the risk of coronary artery disease. In order to increase the reliability of the study results, the causal relationship between selected features and coronary artery disease risk was investigated using five Mendelian randomization methods. They are MR Egger, weighted median, inverse variance weighted (IVW), simple mode and weighted mode. IVW, which assumes that each genetic variant exists independently and can influence outcome only through the exposure of interest and combines the Wald ratios of individual SNPs, was employed as the principal method of analysis in this study. However, causality may be biased in the presence of pleiotropy (Bowden et al., 2015; Grover et al., 2017). The remaining four methods were used as complementary methods to IVW, although they are less powerful (Chen et al., 2020). A statistically significant association between exposure and outcome was deemed to be present when the p-value was found to be less than 0.05.

### 2.7 Sensitivity analysis

Heterogeneity tests were carried out for statistically significant results using Cochran’s Q-test (p < 0.05 was considered heterogeneity). Meanwhile, we used MR-Egger intercept tests to detect pleiotropy (p < 0.05 was considered pleiotropy) (Burgess and Thompson, 2017; Chen et al., 2023). Finally, the leave-one-out sensitivity analysis was performed to examine if one single SNP drove the causal association. In this study, R software and the “Two Sample MR” package were used for all MR analyses.

### 2.8 Statistical analysis

SPSS 25.0 was used for data analysis. For measurement data, the D'Agostino-Pearson omnibus test was first used to assess normality. The measurement data conforming to normal distribution were expressed as mean ± standard deviation ( $\bar{X}$ ± S), and non-normally distributed measurement data were expressed as median (Interquartile range) (M (Q25, Q75), %). The differences between two groups were compared using an independent samples t-test or the non-parametric Mann-Whitney U test. P < 0.05 was considered statistically significant.

## 3 Results

### 3.1 Data exploratory analysis

**FIGURE 1:**
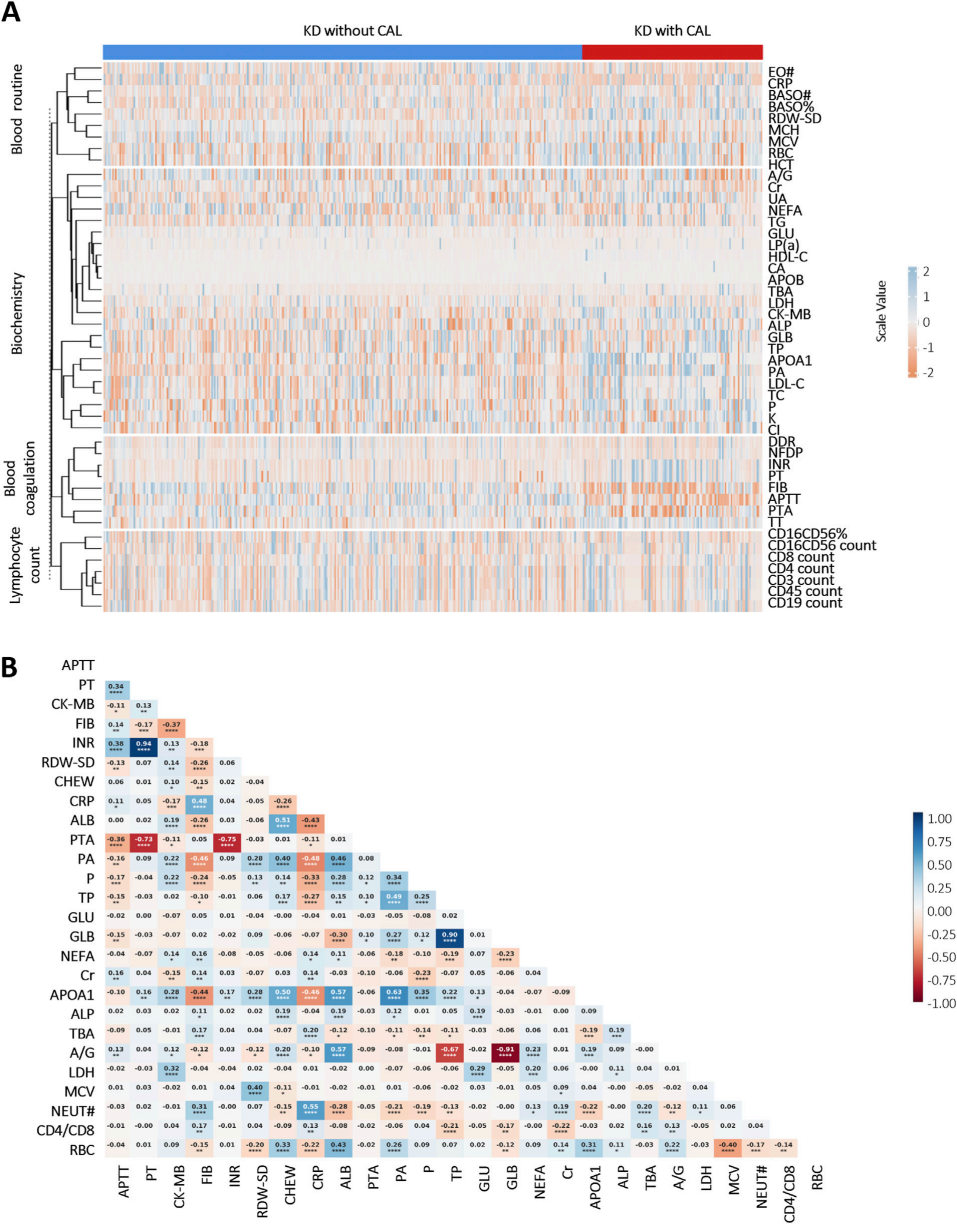
Heatmap of correlation and differential analyses of laboratory biomarkers in KD patients: **(A)** Differential analysis of laboratory biomarkers between KD patients with and without CALs (p < 0.05). Scale values <0 indicate negative correlation differences, and scale values >0 indicate positive correlation differences; **(B)** Correlation analysis of important laboratory biomarkers in children with KD. Colors indicate Pearson Correlation Coefficient (r) between variables—blue for positive and red for negative correlations, with color intensity reflecting the strength of the correlation. The numbers in the cells represent the exact r values, where positive values indicate positive correlations and negative values indicate negative correlations. Asterisks denote statistical significance: *P < 0.05, **P < 0.01, ***P < 0.001, ****P < 0.0001. Abbreviations: Eosinophil count (EO#), C-reactive protein (CRP), basophil count (BASO#), basophil ratio (BASO%), neutrophil (NEUT#), red cell distribution width-standard deviation (RDW-SD), mean corpuscular hemoglobin (MCH), mean corpuscular volume (MCV), red blood cell (RBC), hematocrit (HCT), albumin/globulin (A/G), creatinine (Cr), uric acid (UA), non-esterified fatty acid (NEFA), triglyceride (TG), glucose (GLU), lipoprotein (a) (LP(a)), high-density lipoprotein cholesterol (HDL-C), calcium (CA), apolipoprotein B (APOB), total biliary acid (TBA), lactate dehydrogenase (LDH), creatine kinase-MB (CK-MB), alkaline phosphatase (ALP), globulin (GLB), total protein (TP), apolipoprotein A1 (APOA1), prealbumin (PA), low-density lipoprotein cholesterol (LDL-C), total cholesterol (TC), phosphate (P), potassium (K), chlorine (Cl), D-Dimer (DDR), fibrin degradation products (NFDP), international normalized ratio (INR), prothrombin time (PT), fibrinogen (FIB), activated partial thromboplastin time (APTT), prothrombin activation (PTA), thrombin time (TT).

A comparative analysis delineated multiple laboratory biomarkers between KD patients with and without CALs. The two groups showed significant differences in a variety of indicators (Figure 1A). KD patients with CALs predominantly fell into the high-risk of abnormalities in coagulation system, including activated partial thromboplastin time (APTT), thrombin time (TT) and prothrombin time (PT). We further performed a correlation analysis of laboratory biomarkers in KD patients to explore potential physiological or pathological associations. The results, shown in Figure 1B, illustrate the correlations among several key laboratory biomarkers in KD patients.

### 3.2 Feature selection

**FIGURE 2:**
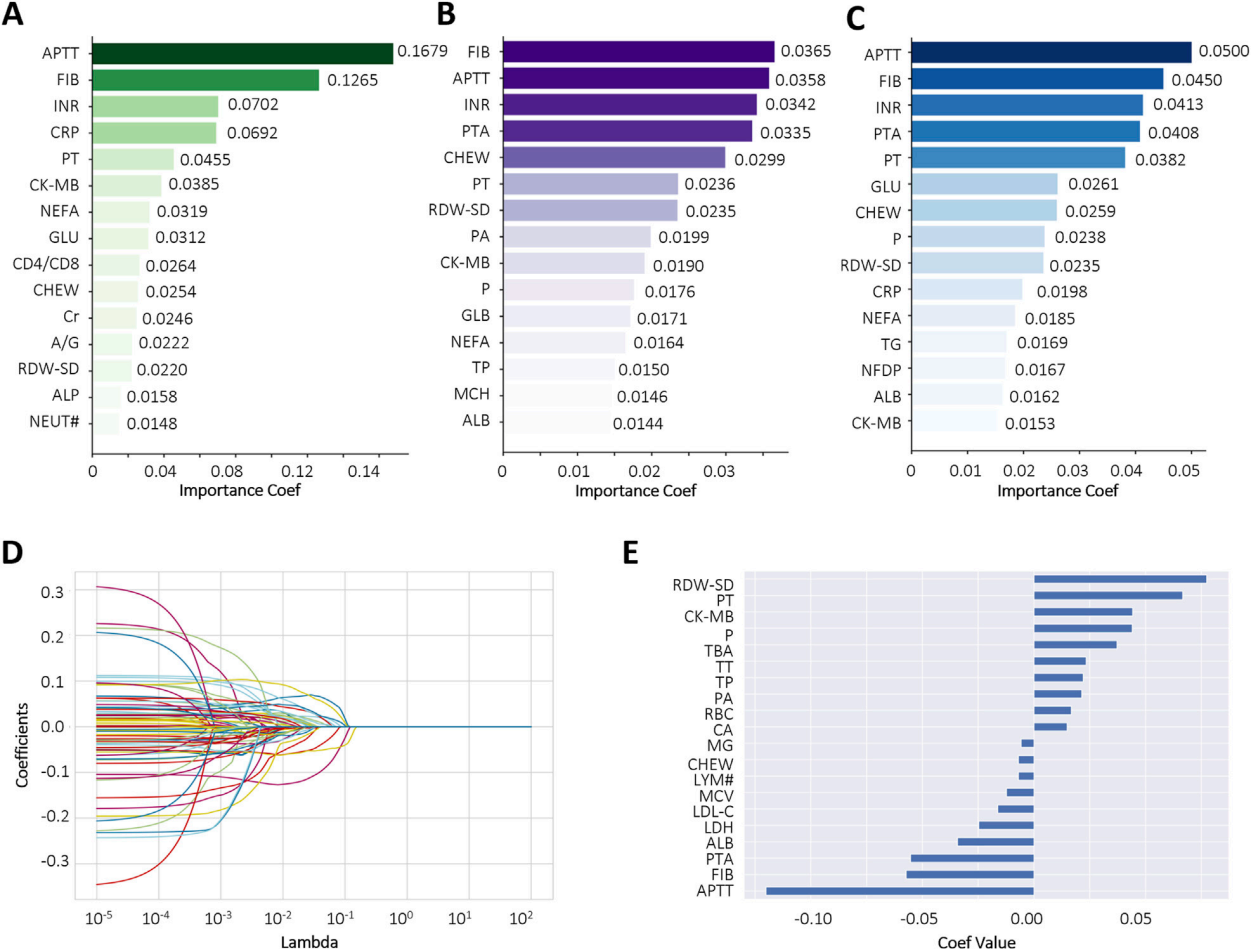
Comparative feature importance rankings across multiple algorithms. **(A–C)** The top 15 most influential features ranked by Gradient Boosting Decision Tree (GBDT), Extra Tree (ET), and Random Forest (RF), respectively; **(D,E)** Feature importance scores derived from LASSO regression using the optimal regularization parameter (best alpha = 0.0098). Each curve represents a biomarker. Abbreviations: Activated partial thromboplastin time (APTT), fibrinogen (FIB), international normalized ratio (INR), C-reactive protein (CRP), prothrombin time (PT), creatine kinase-MB (CK-MB), non-esterified fatty acid (NEFA), glucose (GLU), CD4^+^ count/CD8^+^ count (CD4/CD8), cholinesterase (CHEW), creatinine (Cr), albumin/globulin (A/G), red cell distribution width-standard deviation (RDW-SD), alkaline phosphatase (ALP), neutrophil (NEUT#), prothrombin activation (PTA), prealbumin (PA), phosphate (P), globulin (GLB), total protein (TP), mean corpuscular hemoglobin (MCH), albumin (ALB), triglyceride (TG), fibrin degradation products (NFDP), lymphocyte count (LYM#).

To reduce the risk of overfitting and improve the generalizability of the ML models, a comprehensive feature selection strategy was implemented. The results of feature importance ranking and selection across different classifiers are presented in Figure 2 and Supplementary Tables S1, S2. By integrating the results of ROC analysis with differential expression analysis, we identified a core set of seven key laboratory biomarkers as the most informative for model construction (Table 2). Cholinesterase (CHE) was excluded because it did not reach statistical significance in ROC curve analysis. These selected features included creatine kinase-MB (CK-MB), fibrinogen (FIB), international normalized ratio (INR), APTT, PT, red cell distribution width-standard deviation (RDW-SD), and C-reactive protein (CRP).

**TABLE 2:** ROC Analysis of selected features for distinguishing CAL in KD.

Features	Area	Std. Error	95% confidence interval	p value
APTT	0.7404	0.02812	0.6853 to 0.7955	<0.0001
FIB	0.7380	0.03032	0.6786 to 0.7974	<0.0001
CK-MB	0.6896	0.02985	0.6311 to 0.7481	<0.0001
RDW-SD	0.6754	0.02945	0.6177 to 0.7332	<0.0001
PT	0.6500	0.03303	0.5853 to 0.7148	<0.0001
INR	0.6409	0.03345	0.5753 to 0.7065	<0.0001
CRP	0.5775	0.03059	0.5175 to 0.6374	0.0110
CHE	0.5008	0.03811	0.4261 to 0.5755	0.9796

Note. Activated partial thromboplastin time (APTT), fibrinogen (FIB), creatine kinase-MB (CK-MB), red cell distribution width-standard deviation (RDW-SD), prothrombin time (PT), international normalized ratio (INR), C-reactive protein (CRP), cholinesterase (CHE).

### 3.3 Model development

**FIGURE 3:**
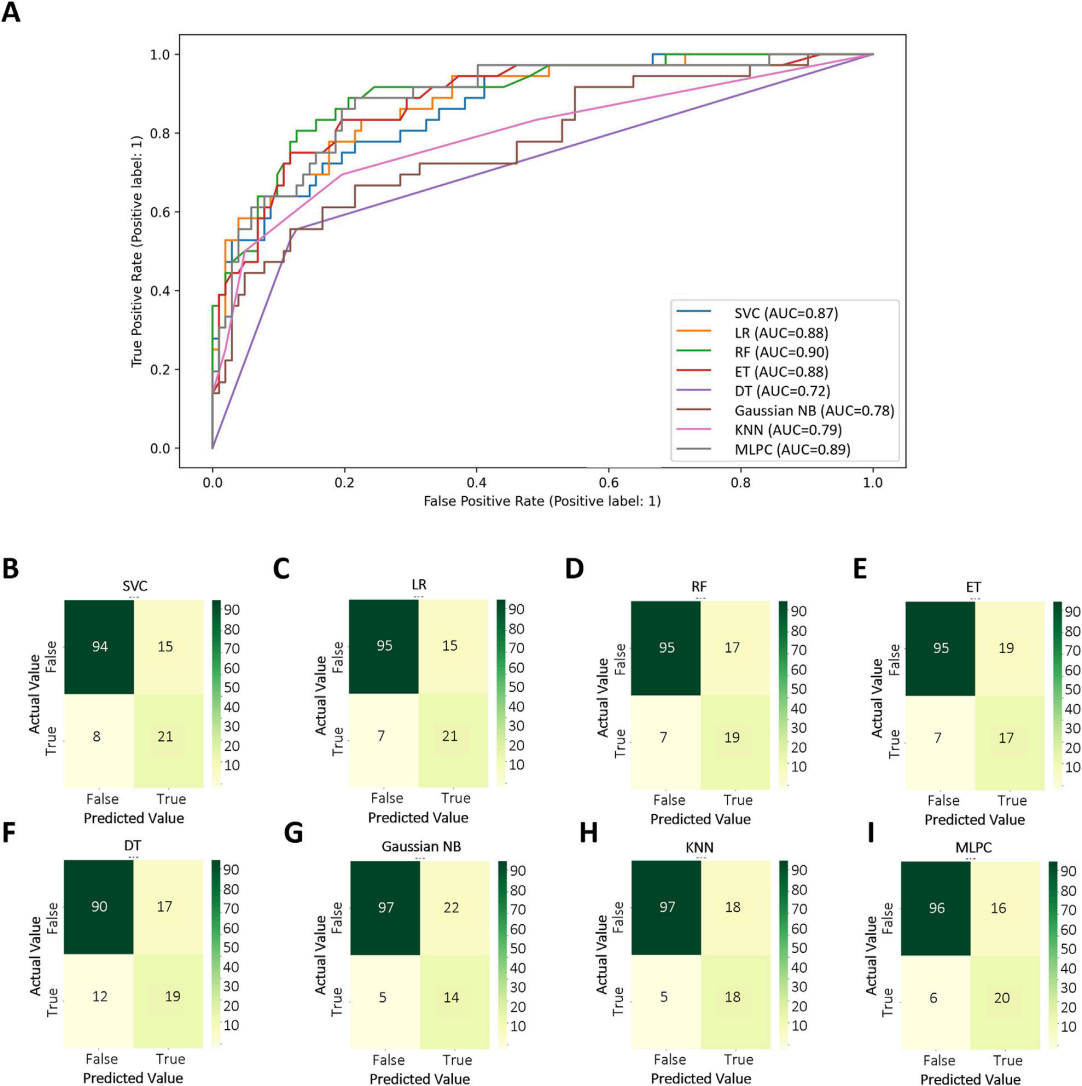
Test set performance of eight machine learning models. **(A)** ROC curves demonstrating the classification performance of each model on the independent test set; **(B–I)** Confusion matrices based on test set predictions, with rows corresponding to the actual labels and columns to the predicted labels. Support Vector Classifier (SVC), Logistic Regression (LR), Random Forest (RF), Extra Tree (ET), Decision Tree Classifier (DT), Gaussian Naïve Bayes (Gaussian NB), K-Nearest Neighbors (KNN), Multi-Layer Perceptron Classifier (MLPC).

Based on selected features, different methods were used in order to get the best CALs risk prediction model. Figure 3 and Table 3 shows the results of the 8 ML models testing. Concerning the whole dataset, the RF model achieved the best performance with AUC of 0.90, whereas most other models gave an AUC above 0.8 (Figure 3A). However, LR and MLPC stand out with the high prediction accuracy 0.84. It means these models have excellent discriminating power in predicting CALs.

These parameters are defined as follows: Precision = True Positive/(True Positive + False Positive), Recall = True Positive/(True Positive + False Negative), Accuracy = True Positive + True Negative /(True Positive + True Negative + False Positive + False Negative), F1-score = 2×Precision × Recall/(Precision + Recall).

**TABLE 3:** Model results of testing dataset.

Model	KD without CALs				KD with CALs				Accuracy
	Precision	Recall	F1-score	Support	Precision	Recall	F1-score	Support	
SVC	0.92	0.86	0.89	109	0.58	0.72	0.65	29	0.83
LR	0.93	0.86	0.90	110	0.58	0.75	0.66	28	0.84
RF	0.93	0.85	0.89	112	0.53	0.73	0.61	26	0.83
ET	0.93	0.83	0.88	114	0.47	0.71	0.57	24	0.81
DT	0.88	0.84	0.86	107	0.53	0.61	0.57	31	0.79
Gaussian NB	0.95	0.82	0.88	119	0.39	0.74	0.51	19	0.80
KNN	0.95	0.84	0.89	115	0.50	0.78	0.61	23	0.83
MLPC	0.94	0.86	0.90	112	0.56	0.77	0.65	26	0.84

Note. Support Vector Classifier (SVC), Logistic Regression (LR), Random Forest (RF), Extra Tree (ET), Decision Tree Classifier (DT), Gaussian Naïve Bayes (Gaussian NB), K-Nearest Neighbors (KNN), Multi-Layer Perceptron Classifier (MLPC).

### 3.4 Model performance

**FIGURE 4:**
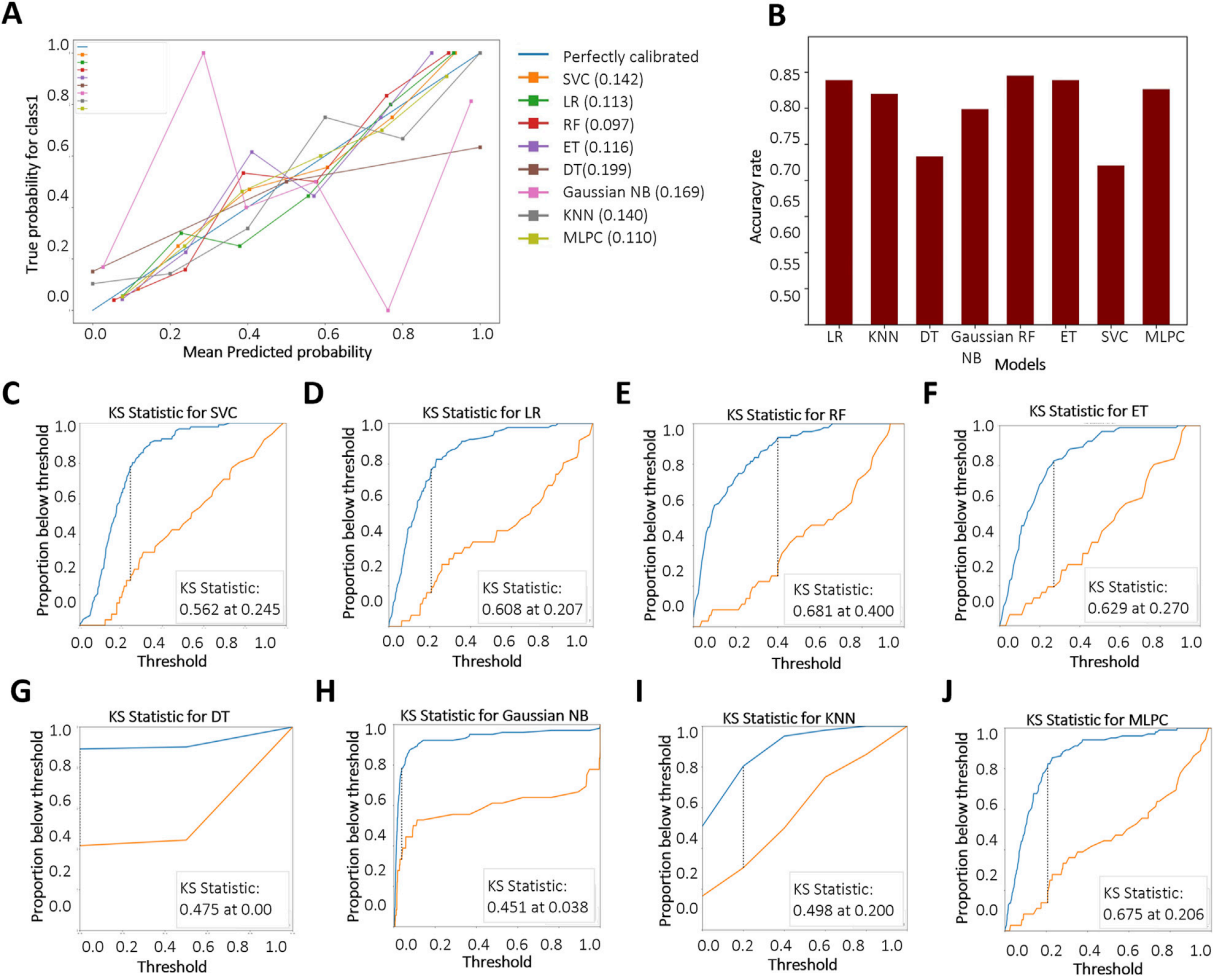
Evaluation and comparison of model performance. **(A)** Reliability curves based on Brier score calculations. “Blue” and “Red” represent KD patients without and with CALs, respectively. Brier scores range from 0 to 1, with lower values indicating better probabilistic calibration; **(B)** Accuracy of each model based on K-fold cross-validation; **(C–J)** Kolmogorov–Smirnov (KS) curves for different models, where a larger separation between the cumulative distributions indicates stronger discriminative ability. Support Vector Classifier (SVC), Logistic Regression (LR), Random Forest (RF), Extra Tree (ET), Decision Tree Classifier (DT), Gaussian Naïve Bayes (Gaussian NB), K-Nearest Neighbors (KNN), Multi-Layer Perceptron Classifier (MLPC).

We further evaluated the models using the BS to assess the accuracy of probabilistic predictions. Among all models, RF, MLPC, and LR achieved the lowest BS, with values of 0.097, 0.110, and 0.113, respectively (Figure 4A), indicating superior probability calibration. Additionally, we applied K-fold cross-validation to compare model performance based on average test error. The top-performing models achieved a training accuracy converging around 0.8. RF demonstrated the highest overall accuracy on the test set (84.5%), followed by LR (83.9%) and ET (83.9%) (Figure 4B). The KS test results are shown in Figures 4C–J. A larger KS statistic indicates stronger discrimination between positive and negative classes. Interestingly, while RF performed well in overall accuracy, it showed relatively poor discrimination capability based on the KS statistic. In contrast, DT and MLPC models yielded KS curves closest to the true distribution, suggesting better class separation performance.

### 3.5 Model validation and web design

**FIGURE 5:**
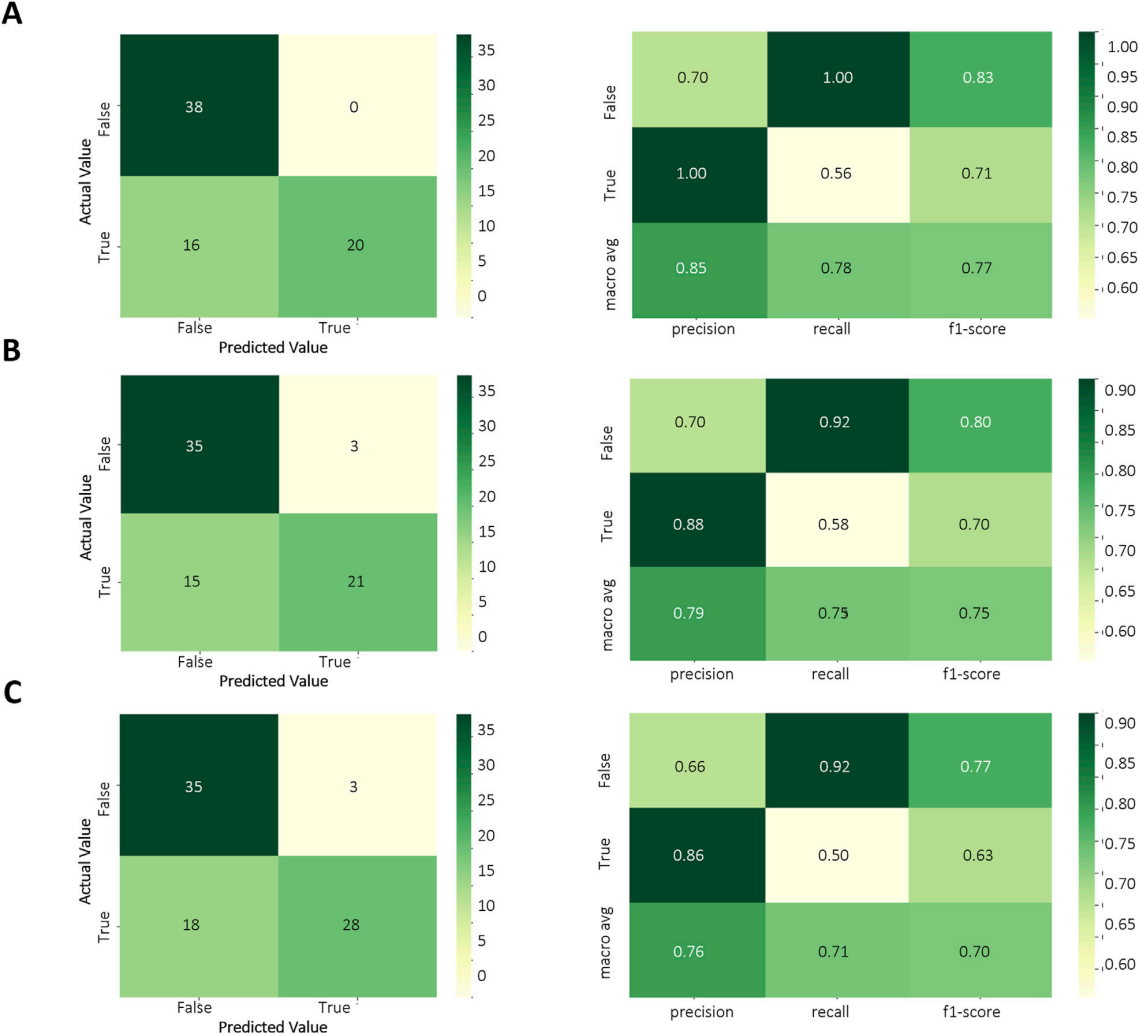
Temporal validation of ML models. **(A)** Confusion matrix of MLPC model; **(B)** Confusion matrix of ET model; **(C)** Confusion matrix of RF model.

Based on the results of the above model evaluation, we selected three models with better performance for data validation. They were MLPC, ET and RF. We proposed a time-cross validation in total of 74 KD patients collected from future (2022–2023), including 36 patients with CALs and 38 patients without CALs. The external validation results were shown in Figure 5 and Table 4. The accuracy of MLPC is 0.78, ET is 0.76, and RF is 0.72. Therefore, we chose MLPC as our final prediction model. To facilitate the use of our prediction models, we developed this model (http://127.0.0.1:5000).

**TABLE 4:** Time-cross validation.

Model	KD without CALs				KD with CALs				Accuracy
	Precision	Recall	F1-score	Support	Precision	Recall	F1-score	Support	
RF	0.66	0.92	0.77	38	0.86	0.50	0.63	36	0.72
ET	0.70	0.92	0.80	38	0.88	0.58	0.70	36	0.76
MLPC	0.70	1.00	0.83	38	1.00	0.56	0.71	36	0.78

Note. Random Forest (RF), Extra Tree (ET), Multi-Layer Perceptron Classifier (MLPC).

### 3.6 Mendelian randomization

**FIGURE 6:**
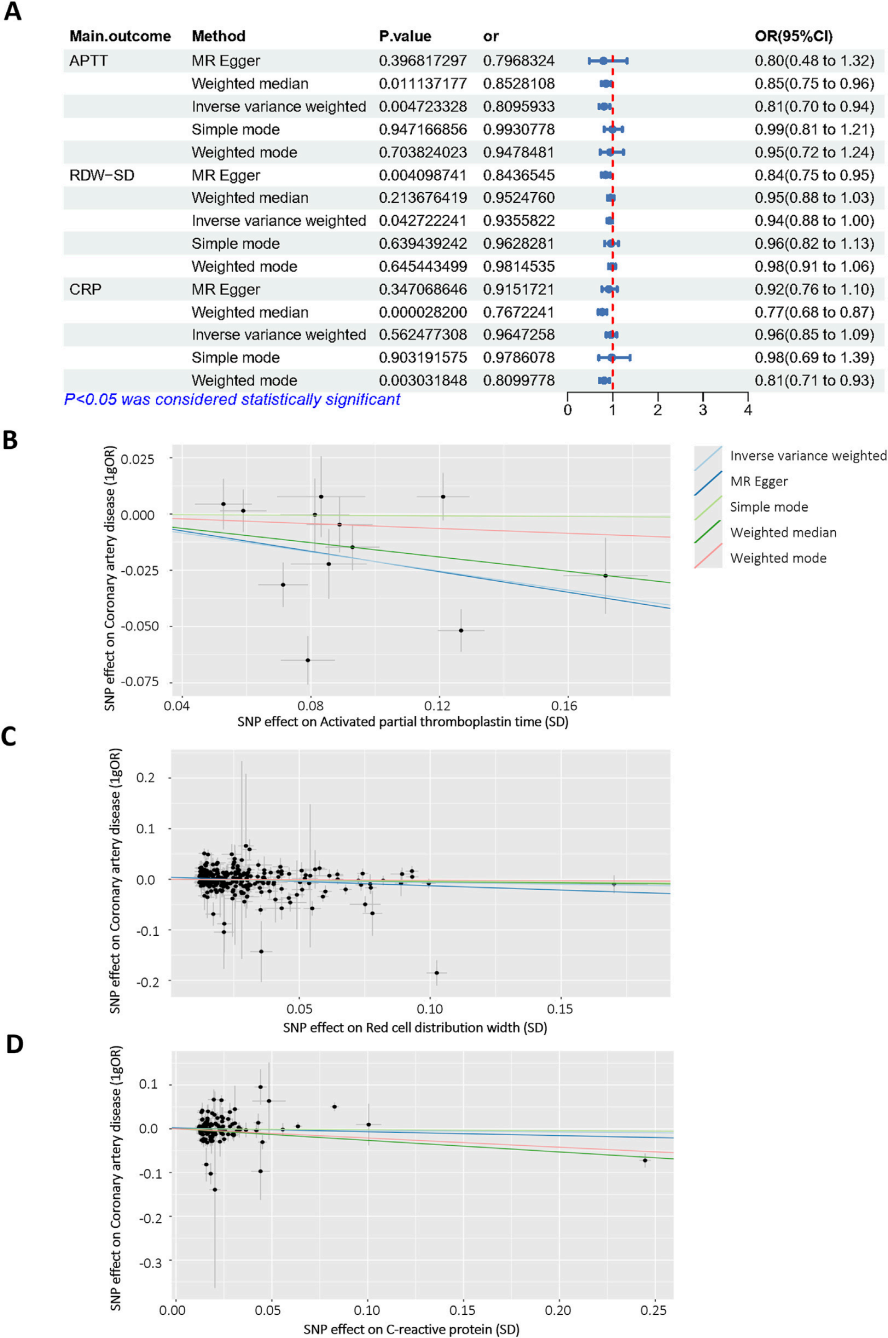
Mendelian randomization analysis of selected features. **(A)** Odds ratio plot of APTT (id: bbj-a-7), RDW-SD (id: ebi-a-GCST9002404) and CRP (id: ebi-a-GCST90018950)/with coronary artery disease (id: bbj-a-159)/. OR: odds ratio; IVW: inverse variance weighted; **(B–D)** Scatter plot of the causal effect of APTT, RDW-SD, CRP on coronary artery disease.

To further confirm the reliability of the model, Mendelian Randomization analysis was performed to confirm the relationship between selected features and CALs. Among all selected features, three features have a clear causal relationship with coronary heart disease. Figure 6A displays the other four additional MR analysis techniques results (MR-Egger, weighted median, simple model, and weighted model). IVW method results revealed evidence of a significant connection between APTT and CALs risk (OR = 0.809, 95% CI = 0.70–0.94, p = 0.004), as well as RDW (OR = 0.935, 95% CI = 0.88–1.00, p = 0.04). This association was further supported by the scatter plot (Figure 6B). Finally, we performed the leave-one-out analysis by removing each instrumental SNP to ensure that no single SNP heavily influenced the causal estimate. Forest plots were generated for each variable (Supplementary Figure S1). No SNP had a substantial effect size on the study’s estimation, indicating the robustness of the findings. The above findings demonstrate a consistent, genetically-based causal relationship between APTT or RDW and CALs.

## 4 Discussion

KD is the leading cause of childhood‐acquired heart disease in the developed world (Miyabe et al., 2019; Miura et al., 2018). It can cause multiple cardiovascular complications, which is known to induce pathological alterations in medium-sized arteries, particularly coronary arteries. Exploring the high-risk factors of coronary injury complicated by KD has always been a research hotspot for scholars around the world, due to the low sensitivity and specificity of the existing research methods (Beltran et al., 2023; Xie et al., 2018; El-Askary et al., 2017). The analysis of clinically related indicators is particularly important. This study retrospectively analyzes the clinical data of 506 children with KD in the Children’s Hospital of Fudan University, and discusses the high-risk factors of KD complicated by CALs. We hope to predict coronary artery injury early, and provide a basis for effective treatment measures.

Recently, an increasing number of studies have been conducted on KD diagnosis using artificial intelligence (AI) (Lam et al., 2022; Kostik et al., 2021). For example, Wang et al. used retrospective retrieval of clinical electronic case information, and then successfully identified KD patients through deep learning algorithms (Wang et al., 2020). In our study, we analyzed a range of laboratory indicators and developed a series of novel predictive models. Our RF model accurately predicts CALs risk in KD patients (AUC: 0.84, accuracy: 80%). It uses routine lab markers (APTT, PT, RDW-SD) instead of advanced imaging, making it practical for emergency or resource-limited settings. The model highlights two key risk drivers: coagulation dysfunction (prolonged APTT, elevated FIB) and systemic inflammation (RDW-SD, CRP). APTT has been used to evaluate endogenous coagulation pathway (Depasse et al., 2021; De Vries et al., 2019; Oskarsdottir et al., 2021). Recent evidence suggests that coagulation-related indicators may be promising markers for the diagnosis of cardiovascular disease (Slack et al., 2022). RDW has been shown to be significantly associated with CRP and cardiovascular disease mortality. The greater the RDW in patients with acute myocardial infarction, the greater the likelihood of another major adverse cardiovascular event within 1 year (Li and Xu, 2023).

According to the obtained results, 8 models were impressive with an average accuracy of 0.80. MLPC, ET and RF models stand out with the high AUC values and favorable accuracy in classification between KD with or without CALs. We also made a time-cross validation study to verify the models’ performance. MLPC had the highest accuracy, so we chose it as our final prediction model. MLPC is a feedforward artificial neural network model that maps multiple input data sets to a single output data set. It can handle nonlinear relationships and has good fitting ability. Features can be extracted automatically, reducing the effort of manual feature engineering (Li et al., 2019; Chen et al., 2018). Although big data is often required, MLPC has the advantage of being able to learn complex interactions through hidden layers. Moreover, we are able to adjust the network structure and parameters to make it more suitable (Dimitriadis et al., 2018; Guo et al., 2020; Ueno et al., 2020). This feature gives it the possibility that it can be implemented even in different hospitals in different regions.

Beyond merely building models, we also applied univariable MR methods, using the largest number of SNPs identified from the latest GWAS for APTT, RDW- SD, FIB, PT, CK-MB and coronary artery disease. Among the 7 features, APTT, RDW, CRP exhibited strong correlation with coronary artery disease. The acquired results corresponded to our data and confirm the reliability of the model. Studies which have used genetic variation in coronary disease genes do give some support to our findings (Aragam et al., 2022). This dual-validation framework (ML + MR) enhances clinical confidence in the model’s predictions and establishes a paradigm for integrating AI with genetic epidemiology in pediatrics.

A strength of our work is the universal availability of features and the time-across validation. Although the gold standard for CALs diagnosis is echocardiographic findings, it would not be readily available in an emergency room. Our model uses routinely ordered laboratory studies and assessable clinical features, making it an effective screening tool at the point of initial evaluation before more costly testing is ordered.

We also recognized the limitations of our work due to the lack of multicenter sites data for external validation. Besides, the current algorithm is only optimised for laboratory test values collected at the time of initial evaluation, and it is unknown how it would perform with data collected at a later timepoint.

In summary, our study demonstrates that ML models, combined with genetic validation through MR, can effectively predict CALs risk in KD patients. By providing a reliable, interpretable, and clinically actionable tool, this approach has the potential to transform the management of KD. Future work will include retrospective validation in external patients with KD, as well as refining the implementation within the clinical workflow.

## Data Availability

The original contributions presented in the study are included in the article/Supplementary Material, further inquiries can be directed to the corresponding authors.

## Supplementary material

SUPPLEMENTARY FIGURE S1 Funnel plots assessing the causal effects of laboratory biomarkers on coronary artery lesions.
