# Correction: Integrative machine learning and Mendelian randomization identify causal laboratory biomarkers for coronary artery lesions in Kawasaki disease: a prospective study

**DOI:** 10.3389/fgene.2025.1735649

**Published:** 2025-11-12

**Authors:** Hancao Yang, Meng Wu, Keqing Liang, Yi Li, Ran Yang, Beibei Yuan, Ming Wu, Jin Xu

**Affiliations:** 1 Department of Clinical Laboratory, Children’s Hospital of Fudan University & National Children Medical Center, Shanghai, China; 2 Department of Clinical Laboratory, Children’s Hospital of Nanjing Medical University, Nanjing, China; 3 Department of Pediatric Surgery, Children’s Hospital of Fudan University & National Children Medical Center and Shanghai Key Laboratory of Birth Defect, Shanghai, China

**Keywords:** Kawasaki disease, coronary artery lesions, machine learning, Mendelian randomization, laboratory biomarkers

Author Jin Xu was erroneously assigned to **affiliation** “Department of Clinical Laboratory, Children’s Hospital of Nanjing Medical University, Nanjing, China”. This affiliation has now been removed for author Jin Xu.

**Affiliation** “Department of Clinical Laboratory, Children’s Hospital of Fudan University and National Children Medical Center, Shanghai, China” was omitted for author Jin Xu. This affiliation has now been added for author Jin Xu.

The original article has been updated.

