# Supplementary material for: Integrative machine learning and Mendelian randomization identify causal laboratory biomarkers for coronary artery lesions in Kawasaki disease: a prospective study
Source: Front Genet. 2025 Aug 15;16:1646032. doi: 10.3389/fgene.2025.1646032 (PMC12394532; doi:10.3389/fgene.2025.1646032)
Supplement: Supplementary file 1 [file Table1.docx]

**Supplementary Table 1.** Feature importance scores derived from different classifiers.

| **Features** | **Random Forest** | **Gradient Boosting Decision Trees** | **Extra Trees** |
| --- | --- | --- | --- |
| CD3 | 0.005547315893585746 | 3.143957229131128e-05 | 0.0086351037510284 |
| CD3 count | 0.010762901485113394 | 0.000519718686610344 | 0.012118595459861782 |
| CD4 | 0.00806663732896843 | 0.009821534952408884 | 0.008039671059091659 |
| CD4 count | 0.010730636984284708 | 0.00826494332779752 | 0.010548918497797469 |
| CD8 | 0.0047413144347554 | 0.008368440806692334 | 0.008130017394883214 |
| CD8 count | 0.008488680972704006 | 0.0005512297061841099 | 0.00987699048225084 |
| CD19 | 0.004889506625053845 | 0.0004385053048513548 | 0.006572437671580484 |
| CD19 count | 0.007335296604498594 | 0.002002076722394624 | 0.01147444774423205 |
| CD16CD56 | 0.011064883224154283 | 0.0037413236086382214 | 0.010072305414512667 |
| CD16CD56 count | 0.007270799790568693 | 0.0005842289648119601 | 0.009136923587059347 |
| CD4/CD8 | 0.00818545840733238 | 0.026434235677509656 | 0.008057793515808197 |
| CD45count | 0.009603769177162528 | 0.0025923222011663373 | 0.010300043947016876 |
| PT | 0.03815951937368285 | 0.04550164113418708 | 0.023550919035803235 |
| INR | 0.041338706393414686 | 0.07022157709778089 | 0.03415518139746674 |
| PTA | 0.04079720742625606 | 0.006119420823366533 | 0.033546237164245006 |
| APTT | 0.05003353429834722 | 0.16786507214545868 | 0.035830836872870635 |
| FIB | 0.044979432187426546 | 0.12652449668792237 | 0.036539585390671786 |
| TT | 0.013281578130004301 | 0.0023034970599042593 | 0.012474864241015163 |
| DDR | 0.009140026465982536 | 0.007932140893812783 | 0.009873331881812443 |
| NFDP | 0.01666979418558963 | 0.000934887322229346 | 0.009494090691432491 |
| TBIL | 0.009869595041957583 | 0.0017224784035204473 | 0.009774525067203537 |
| DBIL | 0.009824548549126646 | 0.0002900000639479719 | 0.010085128363732083 |
| ALT | 0.008710751073068177 | 0.00036628424625372166 | 0.007117849438816882 |
| AST | 0.011132661735479006 | 0.009304058722320489 | 0.009604335280842088 |
| ALP | 0.009896407095144229 | 0.015838695372640016 | 0.009919608551656436 |
| GGT | 0.003941880917200052 | 0.0025192026995913 | 0.007442263662833498 |
| TBA | 0.010372870896690027 | 0.0011839058178146472 | 0.014177921902925703 |
| CHEW | 0.0259407318542497 | 0.025439650600182124 | 0.02989425082078915 |
| UREA | 0.006550142199068195 | 0.0006550414711111377 | 0.008302209781027406 |
| Cr | 0.008795695672080278 | 0.02457951843895096 | 0.010464482246294803 |
| UA | 0.00567666387711873 | 0.001006989749155618 | 0.00914841155927271 |
| TP | 0.011781910945455595 | 0.007921731106754322 | 0.015019707812860775 |
| ALB | 0.016214216059290184 | 0.012197919360034198 | 0.014417387270145441 |
| GLB | 0.013437072196036973 | 0.008494311013645712 | 0.017123363983112507 |
| A/G | 0.010296283378141636 | 0.02216649558905627 | 0.014284919186018012 |
| PA | 0.015106023411867347 | 0.006662666178209576 | 0.019854247970432476 |
| GLU | 0.026076966133648335 | 0.031203540527932453 | 0.0114659799533342 |
| K | 0.013313811461486516 | 0.009269192757070437 | 0.013861817545956034 |
| NA | 0.008078614370082513 | 0.008763190597886819 | 0.010269422156292955 |
| Cl | 0.010119062631594651 | 1.6838661504917699e-06 | 0.01026185715422576 |
| CA | 0.009065223888985369 | 0.010901874061554056 | 0.013212274347204261 |
| P | 0.02375483909711822 | 0.009575602460022481 | 0.017611921765097776 |
| MG | 0.005326995144765079 | 7.441455081467352e-05 | 0.009933121389089578 |
| TC | 0.005496133457815653 | 0.0019428492140729447 | 0.009362298600835773 |
| TG | 0.016935833463174177 | 0 | 0.010639747294306338 |
| APOA1 | 0.014883885487128655 | 0.013200617934386386 | 0.013594979641809508 |
| APOB | 0.00601746055305452 | 0.00011276926738278973 | 0.009293400416785565 |
| HDL-C | 0.01052343519809383 | 0.003490247262451958 | 0.012365467219228418 |
| LDL-C | 0.010042322826764554 | 0.0002455561801795862 | 0.009817296770913654 |
| LP(a) | 0.010027816114278405 | 0.0060168686841070955 | 0.01024950200608896 |
| NEFA | 0.01847130465926808 | 0.03189604355356094 | 0.01643916159679581 |
| CK | 0.00980766437644623 | 0.0023410249577063644 | 0.01211154677944829 |
| LDH | 0.012139904140874855 | 0.00766758670353242 | 0.007979994300968548 |
| HBDH | 0.006808914258215953 | 0.00020025803241249712 | 0.008915291883070393 |
| CK-MB | 0.015298586613940544 | 0.03850394388033283 | 0.019049832021418696 |
| RBC | 0.00799855070132919 | 0.0058454306571504066 | 0.009842609747041603 |
| HGB | 0.007310418309753707 | 0.0008720475344514025 | 0.009231117229966557 |
| HCT | 0.009407269465182826 | 0.010763976104647457 | 0.008428371902106104 |
| PLT | 0.009405337905112948 | 0.0032572694970821638 | 0.007833174956367492 |
| CRP | 0.01977921396843603 | 0.06915472290796812 | 0.01415060315733825 |
| WBC | 0.010533362822485834 | 0.009429097069623086 | 0.010602787649743432 |
| P-LCR | 0.006231436586837498 | 0.000320043926510025 | 0.00846791847422763 |
| MXD% | 0.006461980903379352 | 0.002315421414321689 | 0.008876396406480329 |
| MXD# | 0.008303729994004333 | 0.00020198492216399612 | 0.008108516363550347 |
| RDW- SD | 0.023546576385468775 | 0.0220313431718006 | 0.02348216028776715 |
| MCV | 0.008782462853581426 | 0.0017795978893883732 | 0.0108186316710963 |
| LYM% | 0.010637686441594525 | 0.010825687926737094 | 0.011027005429615738 |
| LYM# | 0.013736949646031974 | 0.0023917167895241417 | 0.009308251576775537 |
| MCH | 0.011224909480268538 | 0.0005319698573556682 | 0.014577705962088709 |
| MCHC | 0.01203224462158771 | 0.009980331069715298 | 0.011399918610524673 |
| MPV | 0.002237043977585231 | 0.00012758022076860416 | 0.009769805623423632 |
| BASO% | 0.006455537814342862 | 0.0036969139916296395 | 0.01159616108581841 |
| BASO# | 0.009504488272692558 | 0.006355162708352348 | 0.013351823836714306 |
| EO% | 0.007716262126571933 | 0.0048396633611812795 | 0.011018087090724311 |
| EO# | 0.010791668311008052 | 0.009549024318437424 | 0.010685802837992628 |
| PDW | 0.008756187874058205 | 0.0009284331770334877 | 0.008359656147391875 |
| PCT | 0.009691901407239726 | 0.0006856121801885524 | 0.009141186708077662 |
| NEUT% | 0.009454599334423332 | 0.0028206032481869624 | 0.009024525423291654 |
| NEUT# | 0.00917695262942708 | 0.014787420035017764 | 0.009403960880601374 |
