# Supplementary figures and images for: Integrative machine learning and Mendelian randomization identify causal laboratory biomarkers for coronary artery lesions in Kawasaki disease: a prospective study
Source: Front Genet. 2025 Aug 15;16:1646032. doi: 10.3389/fgene.2025.1646032 (PMC12394532; doi:10.3389/fgene.2025.1646032)

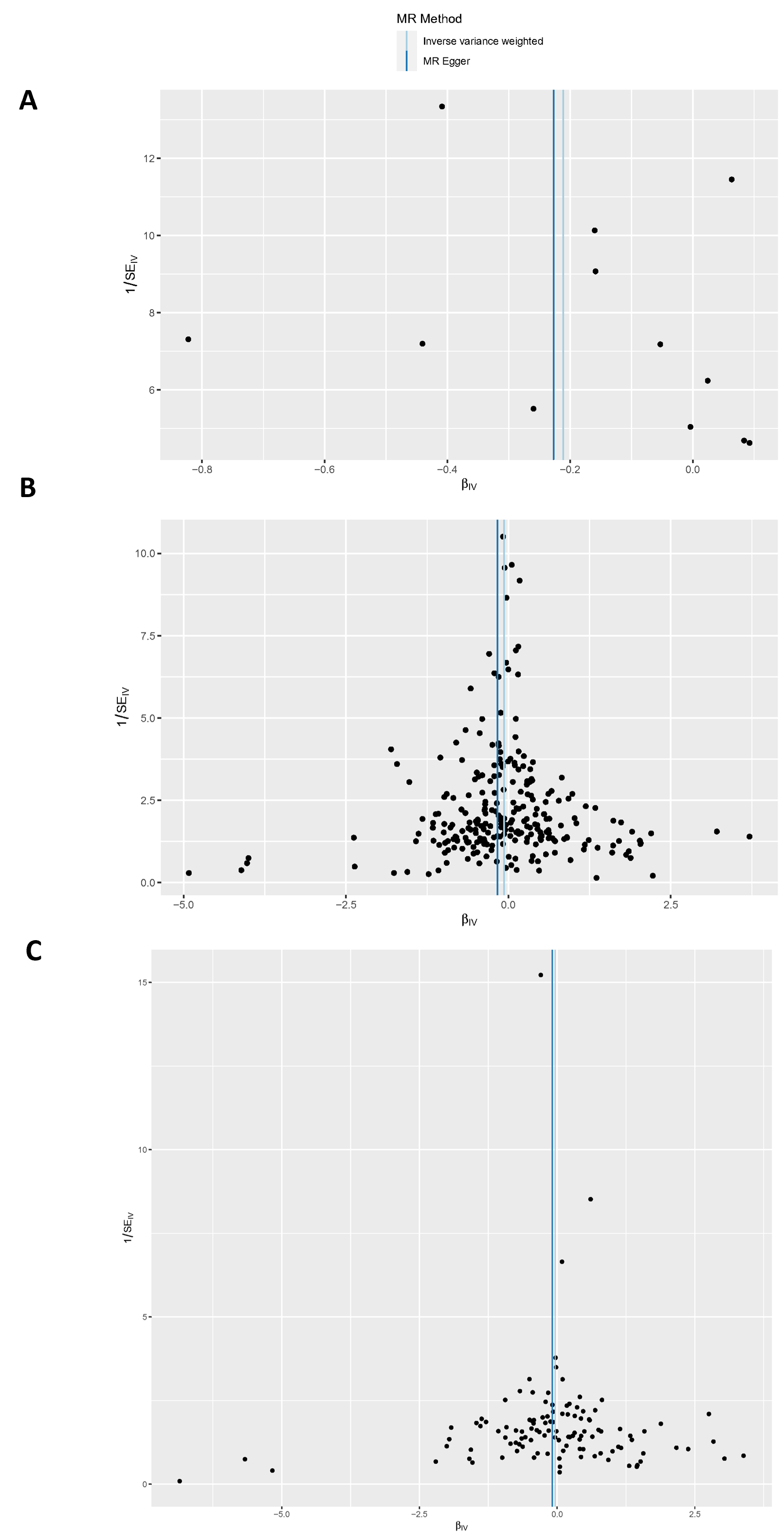

Supplement: Supplementary file 2 [file Image1.tif]
