# Supplementary material for: Integrative machine learning and Mendelian randomization identify causal laboratory biomarkers for coronary artery lesions in Kawasaki disease: a prospective study
Source: Front Genet. 2025 Aug 15;16:1646032. doi: 10.3389/fgene.2025.1646032 (PMC12394532; doi:10.3389/fgene.2025.1646032)
Supplement: Supplementary file 3 [file Table2.docx]

**Supplementary Table 2. Features screened by different classifiers.**

| **Extra Trees** | **Logistic Regression** | **Random Forest** | **Gradient Boosting Decision Trees** | **LASSO** |
| --- | --- | --- | --- | --- |
| PT | CD3 | PT | CD4/CD8 | PT |
| INR | CD4 count | INR | PT | INR |
| PTA | CD19 | PTA | INR | PTA |
| APTT | CD19 count | APTT | APTT | APTT |
| FIB | PT | FIB | FIB | FIB |
| TBA | PTA | NFDP | ALP | TT |
| CHEW | APTT | CHEW | CHEW | ALP |
| TP | FIB | Cr | Cr | TBA |
| ALB | DDR | TP | A/G | CHEW |
| GLB | ALT | ALB | GLU | TP |
| A/G | GGT | GLB | APOA1 | ALB |
| PA | TBA | A/G | NEFA | PA |
| GLU | CHEW | PA | CK-MB | NA |
| P | TP | GLU | CRP | CA |
| TG | ALB | K | RDW- SD | P |
| APOA1 | GLB | CA | NEUT# | MG |
| NEFA | GLU | P |  | APOB |
| CK-MB | K | TG |  | HDL-C |
| CRP | P | APOA1 |  | LDL-C |
| RDW- SD | LDH | NEFA |  | NEFA |
| MCH | HBDH | CK |  | LDH |
| MCHC | CK-MB | CK-MB |  | CK-MB |
| NEUT# | CRP | CRP |  | RBC |
|  | P-LCR | RDW- SD |  | PLT |
|  | RDW- SD | MCHC |  | CRP |
|  | LYM% |  |  | RDW- SD |
|  | LYM# |  |  | MCV |
|  | BASO# |  |  | LYM# |
|  | PDW |  |  | MCH |
|  | NEUT% |  |  |  |

Note. The importance of each feature decreases from top to bottom. Creatine kinase-MB (CK-MB); fibrinogen (FIB); international normalized ratio (INR); red cell volume distribution width (RDW); cholinesterase (CHE); C-reactive protein (CRP); prothrombin activity (PTA); phosphates (P); total biliary acid(TBA); total protein(TP); prealbumin(PA); albumin(ALB); nonestesterified fatty acid (NEFA); globulin(GLB); glucose(GLU); the ratio of CD4^+^T cell/CD8^+^T cell(CD4/CD8); creatinine(Cr).
